# Beyond treatment of chronic pain: a scoping review about epidural electrical spinal cord stimulation to restore sensorimotor and autonomic function after spinal cord injury

**DOI:** 10.1186/s42466-023-00241-z

**Published:** 2023-04-13

**Authors:** Antonia Luz, Rüdiger Rupp, Rezvan Ahmadi, Norbert Weidner

**Affiliations:** 1 Spinal Cord Injury Center, Heidelberg University Hospital, Schlierbacher Landstrasse 200a, 69118 Heidelberg, Germany; 2 Department of Neurosurgery, Heidelberg University Hospital, Heidelberg, Germany

## Abstract

Epidural electrical epinal cord stimulation (ESCS) is an established therapeutic option in various chronic pain conditions. In the last decade, proof-of-concept studies have demonstrated that ESCS in combination with task-oriented rehabilitative interventions can partially restore motor function and neurological recovery after spinal cord injury (SCI). In addition to the ESCS applications for improvement of upper and lower extremity function, ESCS has been investigated for treatment of autonomic dysfunction after SCI such as orthostatic hypotension. The aim of this overview is to present the background of ESCS, emerging concepts and its readiness to become a routine therapy in SCI beyond treatment of chronic pain conditions.

## Introduction

Spinal cord injury either due to a traumatic or non-traumatic cause severely affects sensorimotor function in upper and lower extremities depending on the neurological level of injury and injury severity. In addition, SCI patients present with more or less pronounced autonomic dysfunction including bladder and bowel control as well as cardiovascular malfunction. Beyond these symptoms, which become apparent immediately after injury, secondary disease conditions such as neuropathic pain evolve, which in addition affect the wellbeing and quality of life in SCI patients.

In people with acute incomplete SCI and a high potential for neurological recovery, medical (causal treatment of acute non-traumatic causes, decompression surgery in acute traumatic SCI) and rehabilitative interventions aim to promote sensorimotor improvements and restoration of grasping/reaching and standing/walking function. In people with sensorimotor complete SCI and limited spontaneous neurological recovery, the aim of rehabilitation is to compensate for the permanently lost voluntary function by assistive devices such as wheelchairs or other walking aids. Rehabilitative efforts aiming at improvement of motor function are often compromised by SCI associated complications such as high levels of pain, spasticity or cardiovascular dysfunction such as orthostatic hypotension or autonomic dysreflexia [1].

Worldwide, intensive research activities aim to develop restorative therapies, which foster regeneration of the damaged/injured spinal cord, thus promoting recovery of sensorimotor and autonomic function beyond natural recovery. However, the translation of preclinical results of neuroregenerative approaches to clinical application represents a huge challenge still lacking evidence for efficacy in humans. At the current state the only proven treatments for augmentation of functional recovery are intensive, task-oriented neurorehabilitative therapies activating neural plasticity at different levels of the central nervous system [2]. Although clinically relevant functional improvement can be achieved with neurorehabilitation, its effect size is limited, which explains the need for adjunct neuromodulatory interventions.

Spinal cord stimulation has the potential to amplify the effects of activity-based targeted rehab interventions [3]. It represents one of the most advanced concepts in respect to successful translation towards routine clinical use. In recent years, a number of clinical studies reporting substantial effects of epidural electrical spinal cord stimulation (ESCS) on partial restoration of sensorimotor function in people with incomplete SCI have been published. Remarkably, even in the most challenging population—people with chronic and complete SCI—ESCS has shown the potential to facilitate non-voluntary standing and stepping movements [4–10]. The aim of this review is to provide a structured overview of the available clinical data regarding the application of ESCS to promote clinically meaningful neurological and functional improvement following SCI.

## Methods

For this scoping review we conducted a systematic literature search within Medline and Cochrane as well as the Nature Medicine library. The following keywords and MeSH terms were chosen and applied in different combinations: “epidural stimulation”, “paralysis”, “spinal cord injury”, “paraplegia”, “quadriplegia”, “tetraplegia”, “locomotion”, “autonomic function”, “bladder function”. This scoping review followed the methodological framework described in the PRISMA guidelines (http://www.prisma-statement.org) for conducting a scoping study [11, 12]. Moreover, the checklist of the Preferred Reporting Items for Systematic Reviews and Meta-Analyses extensions for Scoping Reviews (PRISMA-ScR) was used [13]. This scoping review was not registered and no review protocol was produced. This review starts with a brief introduction of the pathophysiological principles of ESCS, which were mostly investigated in preclinical studies. The main focus of this review is on clinical studies reporting effects of ESCS on lower and upper extremity sensorimotor function as well as on autonomic dysfunction after SCI (Table 1). To provide a comprehensive overview of all indications for ESCS in SCI, its rationale and efficacy in chronic pain conditions will also be described.

**Table 1:** Clinical studies investigating ESCS

Included studies	Number of participant(s)	Level of injury and ASIA grading	Main functional outcomes	Study type	Level of evidence*
Harkema et al. [8]	1	C7 (AIS B)	Full weight-bearing standing with assistance during ESCS	Case report	4
Angeli et al. [4]	4	T4 (AIS A); T4 (AIS A); C5 (AIS B); T1 (AIS B)	Walk overground with parallel bars (2/4); taking steps on treadmill with body-weight support (other 2/4); standing with trunk stability	Case series	4
Gill et al. [7]	1	T6 (AIS A)	Independent standing; stepping on treadmill; stepping overground with walker and assistance	Case report	4
Rejc et al. [10]	4	T4 (AIS A); T4 (AIS A); T2 (AIS B); C7 (AIS B);	Full weight-bearing standing with self-balance assistance	Case series	4
Rowald et al. [14]	3	T4 (AIS A); T3 (AIS A); T7 (AIS B);	Overground walking with body-weight support after three days of ESCS; leg movements for cycling and swimming; improved trunk control	Case series	4
Wagner et al. [15]	3	C7 (AIS C); C4 (AIS D); C7 (AIS C);	Overground walking with body-weight support; Increased Lower Extremity Motor Scores (ISNCSCI);	Case series	4
Lu et al. [16]	2	C5 (AIS B); C6 (AIS B)	Increased Upper Extremity Motor Scores (ISNCSCI); Improvement in selfcare-subcategories in the SCIM III	Case series	4
Darrow et al. [6]	2	T8 (AIS A); T4 (AIS A)	Improvement of symptomatic hypotension (1/2); volitional voiding (1/2) with residual volumes; recovery of female orgasm (1/2)	Case series	4
Schieferdecker et al. [17]	5 (4 SCI, 1 Multiple sclerosis)	Not specified. All participants were paraplegic	Decreased urine leakage and incontinence	Retrospective case series	4
Herrity et al. [18]	20 (10/10) in intervention group/usual care group)	Range from C2 to T4 (AIS A/B)	Improved bladder capacity in intervention group (whereas no change in usual care group)	Case control study;cohort study	3b

*Levels of evidence were classified according to the Oxford Centre of Evidence-Based Medicine: Levels of Evidence [19]

## Rationale for spinal cord stimulation

### Epidural spinal cord stimulation to treat chronic pain conditions

**Fig. 1:**
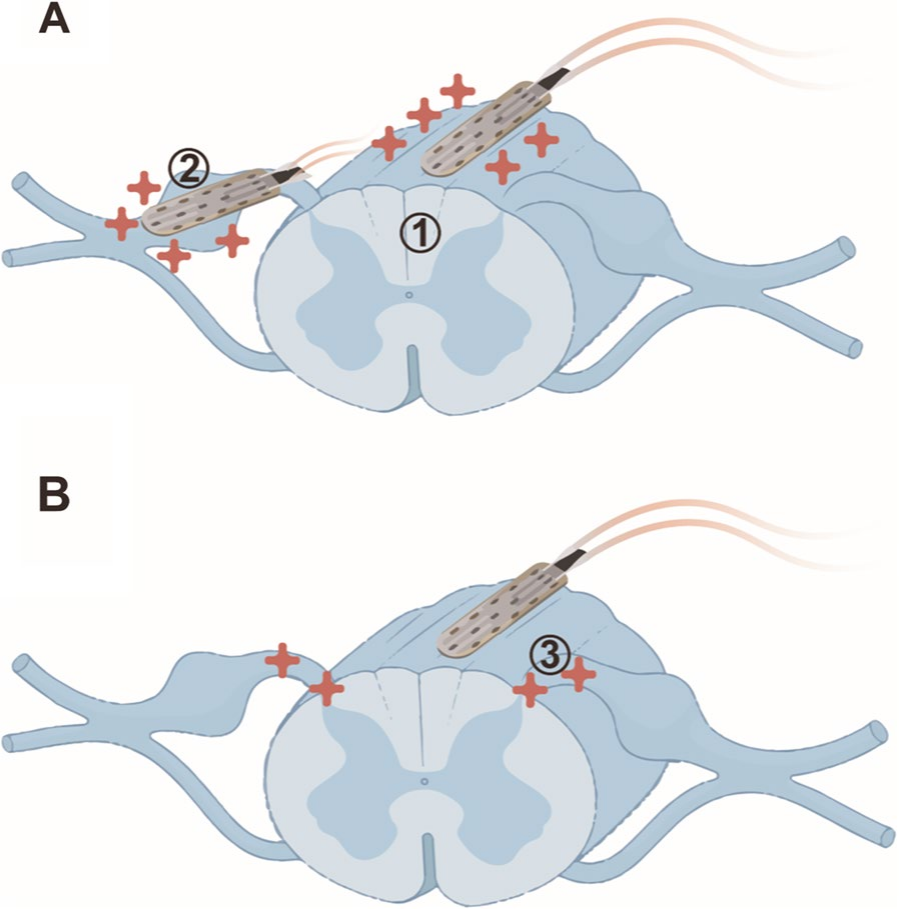
Placement and stimulation target of ESCS. **A** In chronic pain applications electrodes are either positioned epidurally above the lemniscal tract (1) or the dorsal root ganglion (2) to target respective neuroanatomical structures. **B** In case recovery of motor and autonomic function are to be addressed, electrode arrays are placed epidurally above the lemniscal tract to target the posterior roots (3)

The fist clinical use of epidural electrical stimulation of the lemniscal tract at thoracic spinal cord level was described in 1967 targeting chronic pain in a cancer patient [20]. Thereafter, the development of spinal cord stimulators advanced and was especially driven by the company Medtronic Inc. (Dublin Ireland) which received permission for the first fully-implantable ESCS stimulator in 1984 [21]. ESCS was increasingly employed to treat various chronic pain conditions such as neuropathic pain in the extremities, pain after failed back-surgery or complex regional pain syndrome (CRPS) type 1 with electrodes either positioned epidurally above the lemniscal tract or next to the dorsal root ganglion (Fig. 1A). Furthermore, favorable effects of ESCS on spasticity in patients with multiple sclerosis were observed [22, 23].

### Mechanisms of spinal cord stimulation related to locomotor function

In respect to generation of locomotor function following SCI, is was shown in cat models that electrical stimulation directly targeting the vestibulospinal tract [24, 25] or reticulospinal [26] enables hindlimb movements. However, locomotion can also be generated less invasively. Animal studies indicated that ESCS of the lumbosacral spinal cord with absent supraspinal control is capable to induce locomotor patterns by stimulating sensory projections into the spinal cord. It has been shown in quadrupedal animals (mice, cats) that after structurally complete SCI (spinal cord transection) with interruption of all neuronal connections between brain and spinal cord and the associated complete loss of motor control caudal to the level of injury locomotor activity can be facilitated through ESCS. The ESCS-induced effects on locomotor function in mammals are thought to be based on increasing the activity level of spinal circuits consisting of interneurons in the grey matter of the lumbosacral spinal cord commonly referred to as the central pattern generator (CPG) [27]. Activation of the locomotor CPG enables rhythmic alternating coordinated movements of extensor and flexor muscle groups reflecting a walking pattern [28–30]. Somatosensory input plays an essential role in activation of the CPG, thus facilitating the generation of walking patterns. Sensory input shapes and modulates movements to adapt to changing conditions of the environment. In most species, except in cats, absent sensory input leads to uncoordinated and altered motor output or to a discontinuation of movements. Hence, receiving somatosensory and proprioceptive feedback from afferent structures of the skin, joints and muscles of the lower extremities is essential for locomotion [31, 32]. However, animal models have also shown that motor learning deprived of supraspinal input is heavily task dependent. For instance, spinalized cats trained to walk with their hindlimbs improved in locomotion, but not in standing. Vice versa, animals trained to stand failed to regain full walking capabilities [33]. These insights confirm that the “spinal intelligence”, and consequently neuroplasticity within the spinal cord to regain motor function is limited. How exactly motor learning occurs within spinal circuits is still being investigated.

Preclinical studies combined ESCS with the administration of various pharmacological agents [29, 34] thought to strengthen activating neurotransmitter (noradrenaline, serotonin) effects. However, towards clinical development such combinatorial treatments were abandoned due to adverse events caused by these agents in humans.

## Principles of epidural spinal cord stimulation to promote lower limb function

### Neurostimlation basics

The majority of clinical trials applying ESCS in people with SCI used devices originally designed for chronic pain treatment with multielectrode arrays (Medtronic 16 paddle lead) typically placed between vertebral levels of T9 to L1. Although positioned epidurally above the lemniscal tract (Fig. 1B), posterior roots entering the spinal cord at a given spinal segment are targeted [35]. Mathematical simulations and clinical studies showed that large myelinated afferent nerve fiber-derived pseudounipolar dorsal root ganglion neurons as one component of the posterior roots are electrically stimulated by ESCS and activate lower motor neurons in the ventral horn via spinal interneurons [14, 15, 36–38]. Optimal ESCS stimulation parameters are identified individually for each subject. Moreover, the resulting function is dependent of the stimulation frequency, i.e. standing requires different settings than walking. Stimulating the lumbar spinal cord at 25–50 Hz has shown to enable alternating flexion and extension leg movements. Furthermore, tonic stimulation mainly results in leg extension whereas burst stimulation applied in specific sequences can induce alternating movements necessary for locomotion [39]. More recent evidence in humans indicates that the combination of phasic and spatiotemporally differentiated stimulation performs (called targeted EES) better than tonic ESCS [15]. Tonic stimuli bear the risk of antidromic collisions in proprioceptive pathways, which may interfere with physiological sensory feedback important for proper limb positioning [40].

### Neurobiological basics

The main effect of ESCS is that it raises the level of spinal excitability close to generating motor output, which is reduced in people with SCI due to the severely impaired descending spinal pathways. By this increase of spinal excitability, stepping movements can be generated during ESCS even in people with complete lesions. In people with clinically complete lesions, but some preserved descending fibers (so called dyscomplete lesions), supraspinal (voluntary) adaptation of stepping movements can be achieved during ESCS. The improvement of coordinated movements during application of ESCS is most likely not based on structural changes such as axon regrowth, since return of voluntary leg movements has been observed in some patients after just a few stimulation sessions and no long-term recovery of voluntary function without the application of ESCS occurs [5].

## Clinical applications of epidural spinal cord stimulation in people with spinal cord injury

### Clinical studies employing spinal cord stimulation for locomotor function after complete SCI

Previous studies pointed out that high frequency task-oriented training combined with ESCS is the most effective way known today to induce partial recovery of voluntary lower limb function in people with incomplete SCI. However, ESCS was initially applied to restore motor function in people with complete SCI. In 2011, a case study combined ESCS and activity-based therapy in a T1 motor complete, but sensory incomplete (American Spinal Injury Association Impairment Scale (AIS) B) person. Pre-implantation of the ESCS array, the participant received 170 body weight-supported locomotor training and manual facilitation sessions over a period of 26 months. During the training no relevant voluntary electromyography (EMG) activity was detectable. Following implantation, ESCS parameters were individually and iteratively adjusted to achieve standing and stepping function. After a total of 80 sessions (60 min each) of combined activity-based training and ESCS, the study participant was able to stand with minor assistance and full weight bearing for approximately five minutes [8].

Subsequently, case series studies with very small sample sizes were able to replicate and expand on these findings [4, 7, 10]. These trials included up to 4 people with SCI including cervical and thoracic levels of injury. Typically, people with motor complete (AIS A and AIS B) and chronic (> 1 year post injury) SCI were enrolled [4, 7, 8, 10]. ESCS paddle leads were either positioned over low thoracic to high lumbar spinal segments [7, 10] or over high lumbar to high sacral segments [4, 8]. Reported study subjects gained voluntary motor function to a varying degree resulting in full weight bearing and independent standing. For conducting hip and knee flexion, external support was necessary [10]. In one study, two out of four participants gained the ability to walk overground, but with parallel bars for body-weight support. The other two were able to take steps on a treadmill with body-weight support and were able to stand with trunk stability [4].

Correspondingly, stimulation parameters differed among subjects and ranged from 2 to 40 Hz and 1 to 10 V. Stimulation parameters had to be set individually for each subject as the threshold for motor excitability varied substantially and factors such as injury severity or the presence of spasticity had an influence [10]. Studies preferentially used a 16-electrode (5-6-5) array by Medtronic Inc. (Dublin, Ireland). Most study designs incorporated activity-based training sessions before applying ESCS. Training protocols usually consisted of intense balance training, body weight supported treadmill training or stepping with manual assistance by physical therapists. Intensive (pre)training consisted of up to 80 sessions spread over 85 weeks. Another relevant finding is that even when ESCS was applied and the participants were sitting, no EMG activity of muscles of the lower extremities could be detected. This changed as soon as the position was changed from sitting to standing, implying that sensory information resulting from weight-bearing is essential while electrical stimulation alone does not facilitate muscular activation [4, 10].

A recently published study reported about the effects of ESCS employing an electrode array located closely to the location of dorsal roots involved in lower extremity movements in combination with a stimulation program reproducing the natural activation of motor neurons underlying locomotor function in three people with chronic, sensorimotor complete cervical SCI, which are part of an ongoing clinical study (STIMO: Epidural Electrical Simulation (EES) With Robot-assisted Rehabilitation in Patients With Spinal Cord Injury. ClinicalTrials.gov Identifier: NCT02936453). These three study participants achieved stepping function, eventually reaching a walking speed between 0.1 and 0.25 m/s. In the six-minute walk test, the three individuals were able to ambulate 25, 35 and 75 m. Other activities such as cycling and swimming were also reported to be facilitated through ESCS. The outstanding characteristic of this trial is that motor activity could be facilitated already after one day of intervention with ESCS and without a pre-intervention training procedure. Furthermore, the researchers pointed out improved outcomes of spatiotemporal stimulation compared to non-targeted ESCS [14].

Concurrent literature suggests that ESCS for motor complete patients cannot restore clinically meaningful voluntary motor function, but rather serve as a “compensatory” therapy approach to help patients get physically active.

### Clinical studies employing spinal cord stimulation for locomotor recovery after incomplete SCI

In contrast to the limited ambulation outcomes in people with motor complete SCI, the achievable outcomes in people with motor incomplete lesions are seemingly higher. One study involving 3 people with chronic, cervical sensorimotor incomplete SCI (AIS C and D) reported not only of improved locomotor function during the application of widespread spatiotemporal ESCS, but also of long-term gains in volitional control of lower extremity muscles when electrical stimulation was turned off. These improvements were achieved with a combination of ESCS with high-intensity (4–5 2 h-sessions/week) task-specific interventions in particular body-weight supported overground training programs over 5 months. Interestingly, the included participants improved in neurological function over time. For instance, lower extremity motor scores increased in all three people. Moreover, some improvements in motor function, e.g. voluntary control of plegic leg muscles remained even in the absence of electrical stimulation. This might represent one main difference in ESCS application in motor complete vs. motor incomplete people with SCI. ESCS could potentially enhance the effects of task-specific training programs in respect to recovery of motor function in people with motor incomplete SCI (AIS C or D) [15].

### Clinical studies employing spinal cord stimulation for upper extremity function

A cervical SCI results in tetraplegia associated with impaired arm/hand function which can negatively impact the level of independence of the people affected in activities of daily living. These include for example grasping a toothbrush, getting dressed or carrying out transfers. Even partial recovery of volitional arm and hand function are highly desired by people with tetraplegia [41]. Considering the mode of action and therapeutic effects in respect to recovery of lower limb function it is obvious to use ESCS for stimulation of the cervical spinal cord activating spinal neural networks relevant for upper limb function. Preclinical studies in rodents and monkeys following incomplete cervical SCI indicated positive effects on motor recovery [42, 43].

However, to date only one study describes the effects of cervical ESCS on motor function in persons with chronic tetraplegia [16]. Two persons with a motor complete (AIS B) SCI and a neurological level of injury of C5 and C6, respectively, received a 16-electrode array implanted epidurally from C5 to T1 level and connected to a spinal cord stimulator (Boston Scientific, Marlborough, MA, USA) with the primary aim to treat a refractory chronic pain condition. Stimulation parameters included frequencies from 2 to 40 Hz, stimulation amplitudes from 0.1 to 10. mA and a pulse width of 210 µs. Quite remarkably, both study participants gained between 16 and 23 upper extremity motor score points according to the International Standards for Neurological Classification of SCI (ISNCSCI; [44]), which was accompanied by functional improvement, in particular related to the self-care subscore of the Spinal Cord Independence Measure (SCIM) version III [45]. Surprisingly, despite these promising outcomes no other study on cervical ESCS targeting upper extremity motor recovery has been published since. This might be due to surgical challenges placing the electrodes safely within the instrumented spine. However, a few studies using non-invasive transcutaneous spinal cord stimulation reported gains in upper extremity motor function in a small number of people with cervical SCI [46–49].

### Clinical studies employing spinal cord stimulation for autonomic function

SCI can dramatically alter cardiovascular function due to interruption of the autonomic nervous system (autonomic dysregulation), which severely affects the interplay between the sympathetic and parasympathetic nervous system leading to orthostatic hypotension, brady- or tachycardia and/or arterial hypertension. In particular, patients with injury levels T6 and further rostral are prone to autonomic dysregulation, which becomes more pronounced in more rostral injury levels and more pronounced injury severities [50]. Accordingly, fainting and acute cardiovascular events such as stroke/heart attacks can pose severe disease conditions [51]. In particular, early after injury pronounced orthostatic dysfunction can substantially hamper SCI patient mobilization, which represents a key prerequisite for many rehabilitative interventions aiming to improve independence in activities of daily living.

First reports regarding beneficial effects on autonomic function following thoracolumbar ESCS aiming at locomotor function improvement were anecdotal. Not only a direct stabilization of blood pressure in the upright position and increase in heart rate, but also improved temperature regulation and positive effects on whole body metabolism were described (for detailed review see [52]). Only recently, such effects were systematically investigated in studies employing transcutaneous and epidural spinal cord stimulation with special emphasis on blood pressure stabilization. An increase during stimulation between 10 and 40 mmHg was observed resulting in higher tolerance to orthostasis-challenging situations such as moving from supine to sitting or standing position. Of note, placement of the epidural electrodes mostly varied between T10 and S2, usually with electrode positions more rostral to the ones effective for activation of the lumbosacral locomotor interneuron network. Stimulation parameters with frequencies between 15 to 120 Hz, pulse widths of 350 to 450 µs and individually adjusted amplitudes were effective.

Effects regarding temperature regulation and sudorimotor function beyond anecdotal evidence have yet to be confirmed. The same applies to proposed effects of ESCS on other body function controlled by the autonomic nervous system such as bladder, bowel and immune function [6, 17]. A more systematic evaluation of bladder function following ESCS (L1-S1) together with an intense task-specific locomotor training in 10 study participants with chronic SCÍ showed higher bladder volumes reflecting better storage function. However, no improvement in voluntary bladder control was seen [18].

## Safety aspects

A low rate of complications represents a prerequisite for successful use of ESCS in clinical routine. This particularly applies to the use of ESCS in the subacute phase in the first months after SCI. Implantation of the electrode array and stimulator is considered a routine surgical procedure and is established for some decades to treat chronic pain conditions. Although the risk for complications is low, infections and hematomas can occur peri- and postoperatively like in other neurosurgical procedures. The most frequent complication is lead migration, which is typically observed following implantation at cervical level (up to 18% of cases) most likely explained by the higher degree of cervical spine mobility, whereas in the thoracic spine lead migration has been observed in only 7% [53, 54]. Lead breakdown is only rarely observed. In few cases, fibrosis around the electrode array has been described, which may cause secondary cord compression and myelopathy and requires removal of the electrode array [55]. Pain over the site of the implanted stimulator was reported in up to 27% of patients [53, 56]. As pointed out, task-oriented rehabilitative interventions including body-weight supported treadmill training can also bear risks in particular in people with chronic motor complete SCI. For example, in one study investigating a total of 4 people with long-term immobility due to SCI undergoing intensive locomotor and ESCS training, a spontaneous hip fracture was reported [4].

## Conclusion

For chronic pain conditions refractory to conventional pharmacological and non-pharmacological treatments ESCS represents an established therapeutic option. The potential of ESCS for restoration of somatosensory and autonomic function in people with SCI is starting to be exploited.

A number of publications, albeit all referring to non-controlled case series studies, provide proof-of-principle that even in the most severe conditions—sensorimotor complete and chronic SCI – ESCS is capable to at least partially restore lower extremity function such as standing and stepping. In people with preserved motor function below the level of lesion, ESCS together with intensive task-specific rehabilitative interventions might lead to improvements in voluntary motor function such as reaching/grasping and walking beyond levels achievable with task-oriented training alone.

The basic principle of ESCS for restoration of locomotion is based on the stimulation of sensory, mostly proprioceptive input into the dorsal horn of the lumbosacral spinal cord activating the spinal locomotor circuitry and resulting in a coordinated motor output. The main advantage of ESCS for restoration of motor function to direct stimulation of efferent, ventral spinal roots is that ESCS does not cause pronounced fatigue of muscles.

A major task of future ESCS trials is to clearly prove the efficacy of ESCS and generalizability of the outcomes shown in only a few study participants. For this, properly powered, controlled clinical trials are required. For successful integration of ESCS aiming to restore motor function into clinical routine, it is highly desirable to develop systems that can be autonomously operated by the end user to allow for home-based training. Ultimately, end users will decide whether the functional outcome achieved is worth the surgical procedure and the intense physical therapy program.

Another target of ESCS is the improvement of impaired autonomic function, which can severely affect the quality of life of people with SCI. Current research shows that ESCS might be a successful therapy option for orthostatic hypotension. Once evidence regarding benefits of ESCS on autonomic function will be obtained, it still remains to be determined whether this invasive therapy is sufficiently accepted by people with SCI.

## Data Availability

Data sharing is not applicable to this article as no datasets were generated or analysed during the current study.
